# On the complexity of measuring forests microclimate and interpreting its relevance in habitat ecology: the example of *Ixodes ricinus* ticks

**DOI:** 10.1186/s13071-017-2498-5

**Published:** 2017-11-06

**Authors:** Denise Boehnke, Reiner Gebhardt, Trevor Petney, Stefan Norra

**Affiliations:** 1 Institute of Regional Science, Karlsruhe Institute of Technology, Reinhard-Baumeister-Platz 1, 76131 Karlsruhe, Germany; 2 Institute of Geography and Geoecology, Karlsruhe Institute of Technology, Reinhard-Baumeister-Platz 1, 76131 Karlsruhe, Germany; 3 Institute of Zoology, Department of Ecology and Parasitology, Karlsruhe Institute of Technology, Kornblumen Strasse 13, 76131 Karlsruhe, Germany; 4 Institute of Applied Geosciences, Karlsruhe Institute of Technology, Adenauerring 20, 76131 Karlsruhe, Germany

**Keywords:** *Ixodes ricinus*, Forest, Microclimate, Habitat ecology, Meteorological station, Measurement concept, Measurement errors, Standard description

## Abstract

**Background:**

Ecological field research on the influence of meteorological parameters on a forest inhabiting species is confronted with the complex relations between measured data and the real conditions the species is exposed to. This study highlights this complexity for the example of *Ixodes ricinus*. This species lives mainly in forest habitats near the ground, but field research on impacts of meteorological conditions on population dynamics is often based on data from nearby official weather stations or occasional in situ measurements. In addition, studies use very different data approaches to analyze comparable research questions. This study is an extensive examination of the methodology used to analyze the impact of meteorological parameters on *Ixodes ricinus* and proposes a methodological approach that tackles the underlying complexity.

**Methods:**

Our specifically developed measurement concept was implemented at 25 forest study sites across Baden-Württemberg, Germany. Meteorological weather stations recorded data in situ and continuously between summer 2012 and autumn 2015, including relative humidity measures in the litter layer and different heights above it (50 cm, 2 m). Hourly averages of relative humidity were calculated and compared with data from the nearest official weather station.

**Results:**

Data measured directly in the forest can differ dramatically from conditions recorded at official weather stations. In general, data indicate a remarkable relative humidity decrease from inside to outside the forest and from ground to atmosphere. Relative humidity measured in the litter layer were, on average, 24% higher than the official data and were much more balanced, especially in summer.

**Conclusions:**

The results illustrate the need for, and benefit of, continuous in situ measurements to grasp the complex relative humidity conditions in forests. Data from official weather stations do not accurately represent actual humidity conditions in forest stands and the explanatory power of short period and fragmentary in situ measurements is extremely limited. However, it is still an open question to what kind of meteorological data are necessary to answer specific questions in tick research. The comparison of research findings was hindered by the variety of information provided, which is why we propose details for future reporting.

## Background

*Ixodes ricinus* is the main vector of diseases to humans and livestock in Europe. To reduce the disease risk by reliable predictions and management in the future, researchers seek a better understanding of the impact of environmental factors and underlying mechanisms on tick population dynamics. However, ecological field research on the influence of meteorological parameters on a forest-inhabiting species always faces complex interrelations between measured data and actual conditions this species is exposed to. Respective scientific understanding is limited as long as underlying mechanisms are not properly known [1, 2] and if data from official weather stations are used for the analysis, instead of meteorological data from the interior of the habitats [2, 3].

In tick research, the problem of the choice of appropriate meteorological study approaches is even more complex. As shown in Table 1, the usual research approaches rely on very different measurement concepts, different data characteristics and generated parameters [4–10]. To analyze the impact of meteorological parameters on tick populations, these field studies correlate data from tick samplings in forests with data from nearby meteorological stations or occasional measurements at sampling sites. Although every approach certainly was well-considered beforehand, these differences limit comparisons between studies and, as a consequence, the discovering of underlying mechanisms and transferable principles.

**Table 1:** Variability of methods used in tick research to correlate tick and meteorological data

Source	No. of study sites (ticks)	No. of study sites (weather)	Origin of weather data	Origin description	Parameters measured	Height of sensors	Measurement frequency	Weather data (raw data)	Weather data (used in analysis)
[4]	13	13	Measured at study site	Study site (forest)	Ta, Ts, RH	1 m, ground-level, soil-level (Ts)	February-December 2011; single measurement before tick sampling	Point data, 1/month	= raw data
	13	12	Official meteorological stations	Nearby	Ta, RH, prec	n.i.	Continuous measurement	Daily means	Mean of 3 days before sampling
[5]	5	5	Measured at study site	Study site (forest)	Ta, RH, soil water content	5 cm above soil surface (Ta, RH)	May-November 2003; single measurement/weekly	Point data, 1/week	= raw data
	5	1	Meteorological station	Close to the study site	detailed climate data	n.i.	n.i.	n.i.	n.i.
[6]	6	1	Measured at study site	study site (forest)	Ta, RH	1 cm above soil surface (Ta, RH)	March-November 2001–2006; on each visit	Point data (09:00, 10:00 and 11:00 h)	= raw data
	1	1	Meteorological station	Nearby	n.i.	n.i.	n.i.	n.i.	n.i.
[7]	3	2	Official meteorological stations	1 km to highest/lowest study site	Ta, altitude	n.i.	1999–2001 continuous measurement	n.i.	Annual means
[8]	1	1	Meteorological station “Mendeleum”	5 km distance at Lednice	Ta, Ts, RH	1.5 m (Ta, RH), -5 cm (Ts)	1989–2001 continuous measurement	Point data, of the 41 days of tick sampling	Daily min/max values (Ta), Ta, Ts and RH at 7:00 and 14:00 h
[9]	1	1	Automatic station	Located in Neuchatel, 500 m distance	Ta, RH, SD	n.i.	1996–1998 continuous measurement	SD and daily max/ average Ta recorded for each day as a 30-day moving average	Monthly mean of SD, Ta, RH; 5 day mean of Ta (max); means of 29, 16, 9 and 4 days before sampling
[10]	7	n.i.	Environmental Agency of the Republic of Slovenia	Cities close to sampling sites	Ta, RH, SD	n.i.	n.i.	Daily min/max (Ta), daily point data (RH at 07:00 h), SD calculated from Ta and RH at 07:00 h	7-day means of Ta min/max (4 sites) daily/weekly means (Ta), RH (sampling day and one week prior), 7-day mean (SD, week prior to sampling)

*Abbreviations*: *prec* precipitation, *Ta* air temperature, *Ts* soil temperature, *RH* relative humidity, *SD* saturation deficit, *n.i*. no further information given

This goes along with the statement of a group of experienced tick researcher’s, arguing against using data from official weather stations (for statistical analysis of field sampled tick data) and warning the community of resulting misinterpretations [11].

Thus, the question occurs as to which measurement concept(s) should be followed and which information, especially with respect to meteorological data, is necessary to optimize comparability of different studies.

### Motivation and goals

The overall goal of this work was to evaluate the effects of several environmental factors on the density and activity of *Ixodes ricinus* tick populations within the state of Baden-Württemberg, Germany. In this context, this paper addresses three issues: (i) the principal aspects relating to the complexity of forest microclimates and of the resulting analysis; (ii) the microclimatic measurement concept that was specifically developed to address the problem of determining the factors influencing *I. ricinus* density and activity, providing methodological details; this concept was implemented in the form of a large measuring campaign; (iii) demonstration of the measuring errors between forest microclimate and weather data if recorded outside of forests (spatial error) or only occasional (temporal error). Finally, resulting challenges for analysis and conclusions in tick research are discussed.

### Ecology and physiology of *Ixodes ricinus*

*Ixodes ricinus* occurs throughout Europe, with forests as its main habitat [4, 12]. Ticks of this species spend most of their life close to the ground. They rest under leaf litter or in the humus or upper soil layers [6] or quest actively for hosts on vegetation near the ground; this species shows a characteristic pattern in central Europe with an activity peak in spring and a lower peak in autumn [13]. This off-host phase of resting and questing represents 98–99% of the ticks’ life-cycle [14], which is why recent state of research expects environmental conditions in a tick’s microhabitat to be responsible for determining tick population density and the dynamics of questing ticks.

A tick has a certain energy storage (fat content) from the last blood meal, which is successively consumed until it finds a new host or dies [15]. After a blood meal, ticks develop to the next stage. Temperature determines tick development rate and seasonal activity pattern, and is the crucial factor for its northern and altitudinal distribution limits [16–18]*.* Tick survival also depends on sufficient moisture availability in its microhabitat. *Ixodes ricinus* ticks are able to actively absorb water from the surrounding air (an energy consuming process) if the relative humidity is 80–85% or higher, and it loses body water if the relative humidity falls below this [14, 19] or the saturation deficit is too high [9, 20].

A standard tick sampling method is flagging or dragging, which captures a part of the active questing ticks of a study location [11]. Its effectivity depends on, for example, the life stage of *I. ricinus*, questing height and vegetation growth [21, 22]. The total number of active questing ticks, however, represents only a part of the total amount of living ticks in a habitat. The rest is made of ticks which develop after a successful blood meal, replenish water losses, are in dormancy or diapause [23]. The total number of living ticks is seasonally fluctuating, mainly depending on mortality rates and newly hatching larvae [15].

Based on flagged tick data, an initial indication of *I. ricinus* density distribution in forests of the study region of Baden-Württemberg was provided in Boehnke et al. [24]. This model approach has also been expanded to cover all of Germany [25].

### Methodological background

*Ixodes ricinus* ticks are very sensitive to the microclimatic conditions they experience in their forest habitat. One peculiarity of this habitat is that forests shape their own characteristic climate. The canopy reduces solar energy fluxes, wind and precipitation income, and modifies the resulting temperature and humidity conditions [26]. Conditions measured by official weather stations, which are located on open-land at 2 m height [27], are not comparable to the conditions in the forest, and methods of predicting forest data from these open-land data are still missing [3].

**Fig. 1:**
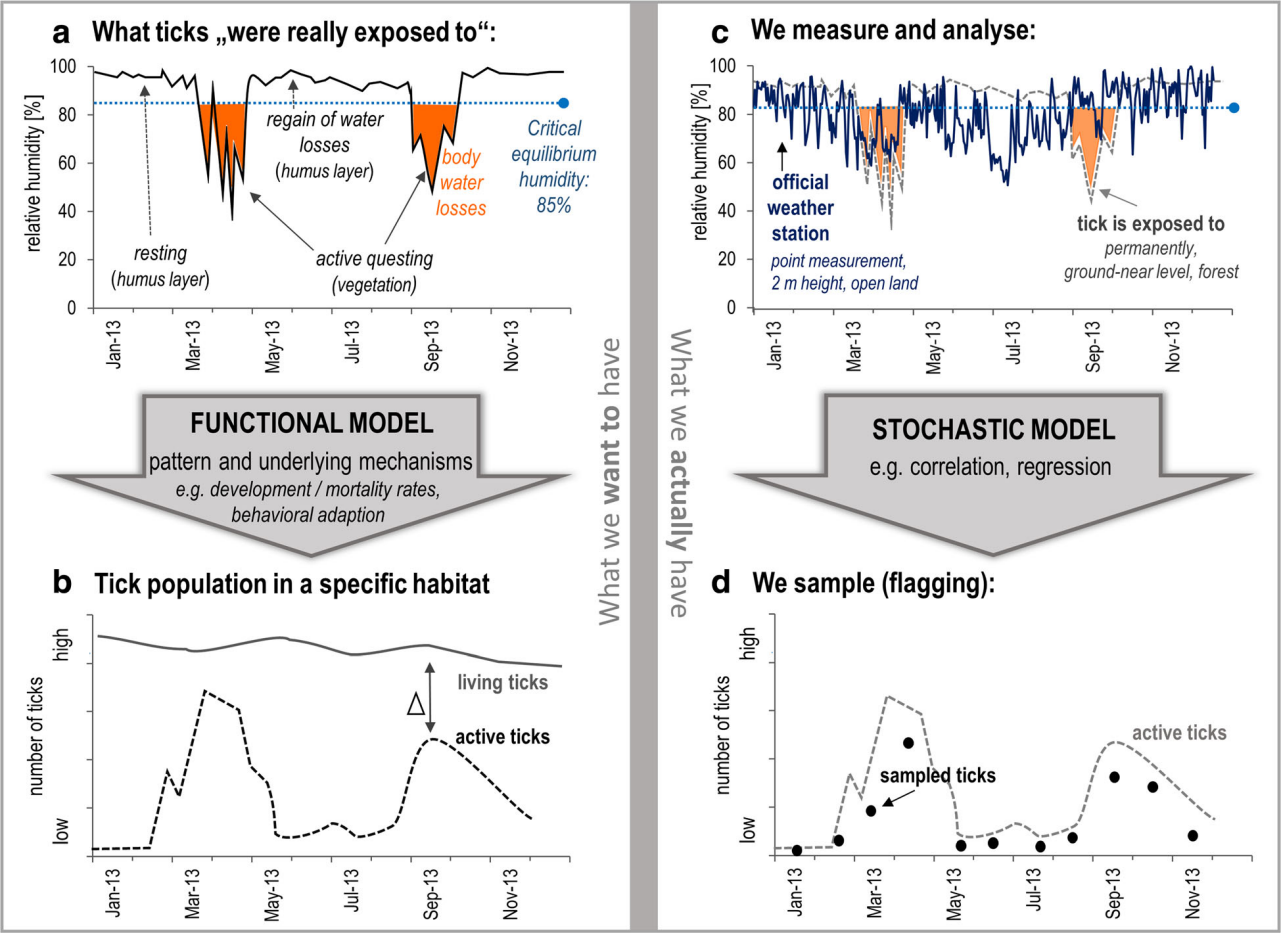
Theoretical considerations about the discrepancy between real conditions and what is represented by collected data. Expected missing reality as a result of complex, ecological interrelations (**a**, **b**) and what we actually capture with our measurement concepts (**c**, **d**) using the example of relative humidity conditions in a tick population’s forest habitat. Graph (**a**) presents a fictive case of the RH conditions ticks were exposed to different situations and highlights water losses (orange) if the humidity falls below a threshold of 85% RH. In contrast, (**c**) illustrates the RH data normally used in tick research, which differ strongly from conditions shown in (**a**) Graphs (**b**) and (**d**) illustrate comparable disparities looking at the tick data; (**b**) gives an impression about the natural discrepancy between the amount of living and active ticks in a habitat, while (**d**) addresses the methodological discrepancy between active and the number of sampled ticks

**Fig. 2:**
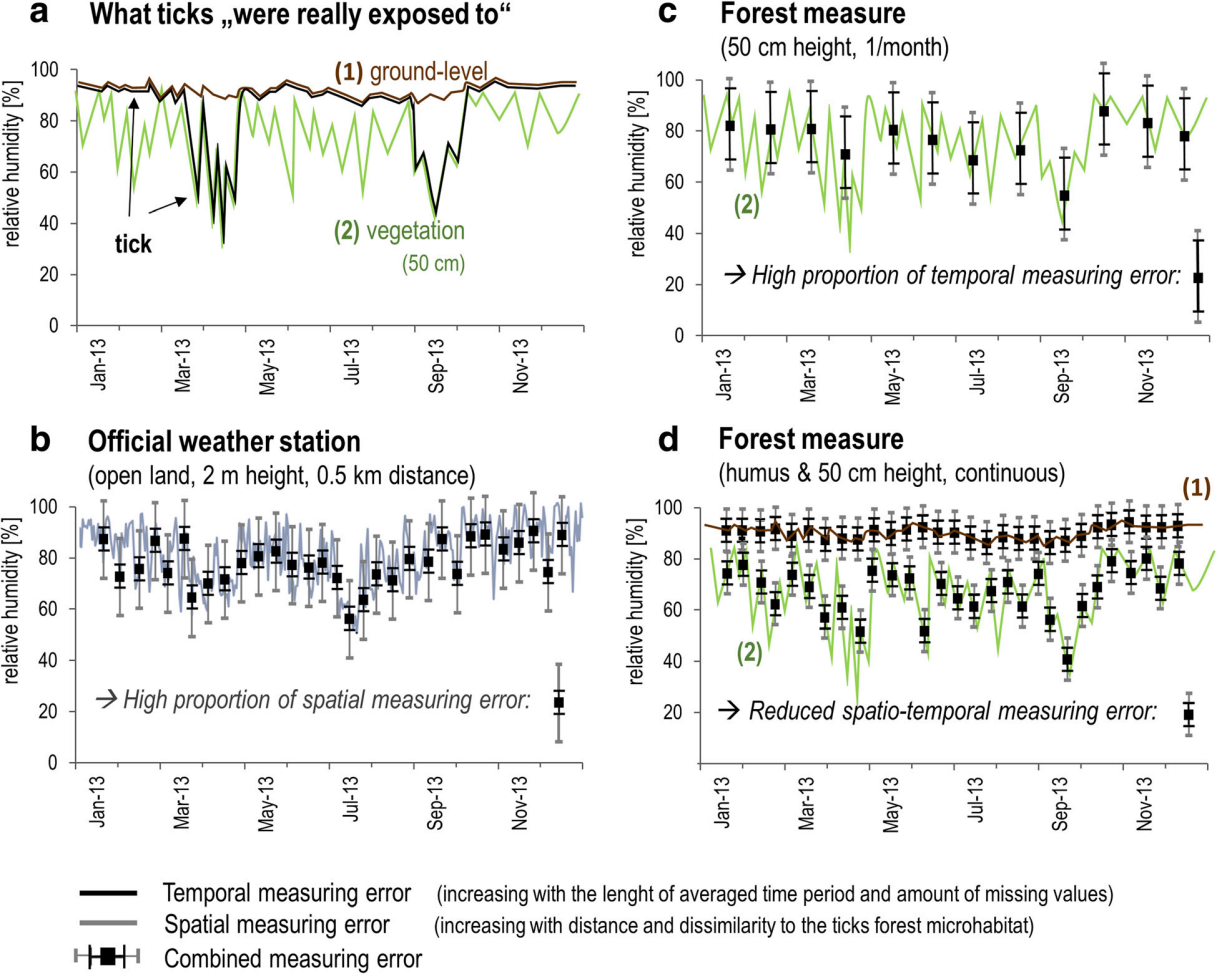
Meteorological data collection methods and connected measuring errors compared to real conditions. A schematic illustration of the measuring errors that exist between real conditions in a tick’s microhabitat (**a**) *brown*: RH conditions at ground-level, *green*: RH conditions in the lower vegetation, *black*: conditions the tick experienced) and data derived from different measuring concepts (**b**, **c**, **d**). Our concept is schematically illustrated in (**b**), with measurements taken at ground-level (error bars on brown line) and in 50 cm height (error bars on green line)

The research presented here involved continuous (micro-) climatic measurements at 25 forest study sites over three years, which currently represents the most comprehensive microclimatic dataset in tick research worldwide. This time- and resource-intensive setup was operated in order to: (i) evaluate the expected discrepancies between the microclimatic conditions ticks are likely to be exposed to in forests and the meteorological data normally used for analysis (Fig. 1) and; (ii) to improve the explanatory power of meteorological data (Fig. 2) and gather new insights into the meteorology dependent ecology of *I. ricinus* ticks. Therefore, this microclimatic dataset offers the perspective of finding relevant new insights in the mechanisms underlying tick distribution patterns (Fig. 1a, b) and contributes to reliable predictions of tick distribution and density in the future [1, 2].

## Methods

### Theoretical aspects

The first part of this section presents a theory on the complexity behind research concerning the influence of microclimate on *I. ricinus* ticks (Fig. 1). It focuses on the discrepancy between conditions (example of relative humidity) ticks actually experience in their microhabitat and resulting consequences for the population on the one hand (Fig. 1a, b) and data used in research to investigate such interdependencies on the other hand (Fig. 1c, d). The second part deals with the relationship between data used for analysis and expected measuring errors. The illustration of existing measuring errors behind different datasets (Fig. 2) demonstrates the benefits of continuous in situ measurements.

In the graphs, the relative humidity values of the official weather station are based on real measurements. The relative humidity in the humus and vegetation is an example of a fictitious forest habitat. The expected physiological reaction of *I. ricinus* is an assumption based on previous findings from. Kahl [14] and Kahl & Knülle [19]. The line of living and active ticks shows how a population in this habitat could react according to existing knowledge [13, 15]. Since the flagging method does not catch all active ticks [21, 22], a lower number of sampled ticks compared to the line of active ticks is assumed.

Fig. 1 schematically illustrates methodological issues in ecological tick research.

The left side shows the humidity conditions ticks were exposed to in 2013 (fictive), the resulting number of living ticks in this habitat and their activity pattern. A physical/functional model links environmental conditions to the resulting tick population, with such a model being necessary for reliable predictions of future tick activity and density. An example of a process-based model is given in [28], which was compiled based on local *Ixodes ricinus* populations and in situ measurements at three British study sites.

On the right side of Fig. 1, the differences compared with the real situation are illustrated, i.e. the measurements rely on official weather stations, a biased underestimate of sampled ticks and an empirical/stochastic model that links the environmental data with the tick sampling.

In detail, Fig. 1a presents a fictive case of the relative humidity (RH) conditions the ticks were exposed to in different situations (resting and rehydrating in the moist humus layer after water loss due to questing in the lower, drier vegetation layer). Simultaneously, consequences for the ticks water balance are illustrated, i.e. water losses if RH falls below the critical equilibrium humidity of about 85% and replacement of those losses if RH is higher than this value [14, 19]. Thus, at present, our knowledge of the microhabitat conditions that ticks really experience, and how those environmental factors affect ticks in the field, is still too limited to predict tick population abundances or activity patterns (Fig. 1b, fictive cases) on the basis of a functional model.

One major problem in field studies is the dissimilarity between the microclimatic conditions that ticks are really exposed to and the data used for analysis. This dissimilarity between “real conditions” (Fig. 1a) and conditions represented by data from an official weather station (data from: German Weather Service, station “Rheinstetten”, data: daily means of RH, 2013-01-01 - 2013-12-31) is illustrated in Fig. 1c.

Another problem is determining the actual number of ticks present (Fig. 1b) based on the number of ticks sampled using the standard flagging method (Fig. 1d) [11, 21]. To improve this data basis, standard flagging data can be combined with data provided by methods with controlled tick numbers (e.g. microcages [29] or arenas [30]). Such research approaches have already provided interesting new findings about the mechanisms underlying tick activity and density.

In the light of these challenges, Fig. 2 illustrates the analytical approach used in this study. Compared to the real conditions (Fig. 2a), we aimed to visualize the complexity of errors caused by an inappropriate data set (Fig. 2b, c) and our measurement concept, aimed at reducing these errors (Fig. 2d).

Fig. 2a schematically illustrates the relative humidity (RH) conditions that the ticks were estimated to have experienced (black line) in a forest in 2013. This very simple estimate based on the observation that the main time of tick questing activity on the vegetation is in spring and autumn and ticks hide near the ground, i.e. the humus, during other periods in central Europe [6, 13]. Conditions were moist during the tick’s phase of resting in the humus (RH conditions at ground level (1), in brown), but were much dryer during questing (RH conditions on the vegetation in 50 cm height (2), in green).

The error types identified and defined in this study are the spatial measuring error (Fig. 2b), which expresses the dissimilarity between actual conditions in a ticks’ forest microhabitat and the data that should represent these conditions, derived from the (official) weather station nearest to the study site but located in open-land. The temporal measuring error describes the dissimilarity between the actual conditions that effect the ticks permanently and occasional measurements (Fig. 2c). This error type increases with the number of missing values and the duration of the averaged period (e.g. annual values) used for correlations, which are both a proxy for the loss of information in the data. Fig. 2d illustrates the potential to reduce the sampling errors as far as possible, and to cover the range of conditions ticks could experience, thereby improving the results of stochastic modelling (Fig. 1c, d).

### Study area

The methods description focuses on the microclimatic setup. Detailed information about all other methods used in the project, especially about the 25 study sites, are given in Boehnke [26].

Baden-Württemberg is located in south-west Germany. It has an area of 35,751 km^2^ and ranges from about 49.783°N (49°47′N) to 47.516°N (47°31′N) and from 7.500°E (7°30′E) in the west to 10.483°E (10°29′E) in the east. The altitude above sea level (a.s.l.) ranges from 85 m (Mannheim) to 1493 m (Feldberg). The climate can be classified using the Köppen-Geiger climate classification as Cfb (C, warm temperate climate; f, fully humid; b, warm summers) [31]. The annual average temperature varies between 3.5 °C and 11.5 °C, the annual average precipitation lies between 600 and 2200 mm within the reference period of 1971–2000, depending on altitude and exposition.

Forests cover 38% of Baden-Württemberg and are mostly restricted to areas unsuitable for other usages (e.g. agriculture). The main tree species are the economically important European spruce (*Picea abies*), with 38% of the total forest area in 2012, followed by European beech, with 21% [32]. Continuous coniferous forests are very common in the Black Forest region. All other regions are characterized by fragments of mixed and deciduous forests surrounded by agricultural and built-up areas [33].

### Study sites and measurement setup

#### Site characteristics

Microclimatic, ecological and tick related aspects were examined at 25 study sites throughout Baden-Württemberg between summer 2012 and autumn 2015. Only forests or forest structures were chosen, since forests are the main habitat of *I. ricinus* ticks in central Europe [4, 12].

Site selection covered various altitudes, average temperatures, average relative humidities and soil types. Thus, the sites included considerable ecological variability. Information about the ecological conditions originated mainly from official governmental websites and is freely available [34, 35]. A short description of the study sites is given in Boehnke et al. [24].

#### Microclimate measurement setup (in situ measurement)

The measurement area comprised the main habitat of *I. ricinus* ticks, i.e. near the soil surface where ticks rest and rehydrate after water loss, and in the lower vegetation where the ticks actively quest for hosts. These data therefore converge to the conditions ticks experience to in their microhabitat. Hence, we expect the data to offer appropriate variables to gather new insights into the relationship between weather conditions and tick population dynamics [1].

The following description is the basis for the presented and future work. The last paragraphs of this section offer details of operational issues to allow detailed methodological reproducibility.

**Fig. 3:**
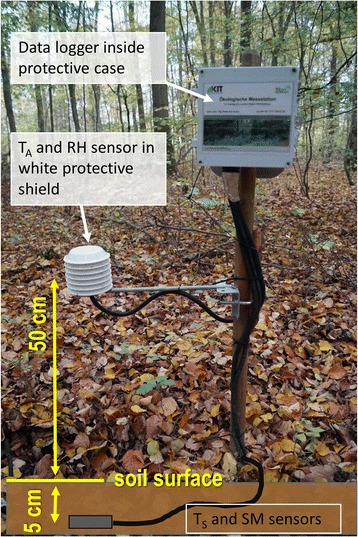
A basic measurement station recording in a deciduous forest

**Fig. 4:**
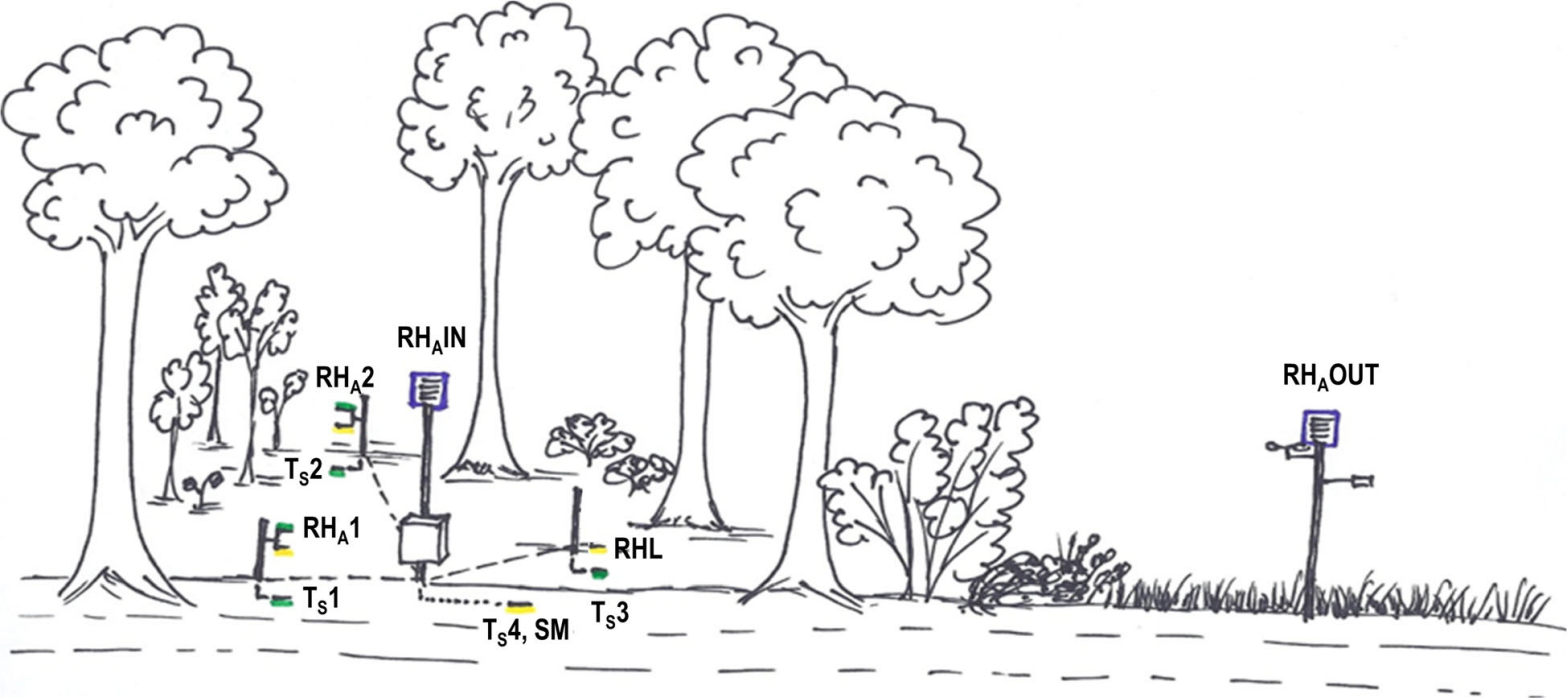
Design of the five intensive measurement stations. Sensors recorded soil-temperature (TS1–4) and soil moisture (SM) at 5 cm depth, relative humidity (and temperature, not labeled) in the litter at 1 cm height (RHL), at 50 cm height (RHA1–2) and at 2 m height inside the forest (RHAIN), and a set of parameters including relative humidity outside the forest (RHAOUT)

At each sampling site, a microclimate station was operated throughout 2013 and 2014 (starting time at different sampling sites: autumn 2012 to spring 2013; end-time at all sites: autumn 2015). Our measurement concept comprised two types of meteorological stations, basic and intensive (Table 2). At basic measurement sites*,* microclimatic measurements focused on the ticks’ basic habitat. We compiled a measurement station for easy and cost-efficient usage, and placed the stations within forest habitats, representing the averaged vegetation structure and species diversity of the forest (Fig. 3). At intensive measurement sites, measurements were expanded to evaluate different microclimatic situations within and nearby the forest, placing the results from the basic sites within a broader context (Fig. 4). Measurements within the forest had a minimum distance to the forest edge of 20 m.

**Table 2:** Configuration of the two types of microclimatic stations used at the 25 study sites

No. of sensors	Parameters measured	Height of sensor	Sensor specification
Basic measurement sites (20 sites)^a^			
1	Ta, RH	50 cm above soil surface	S-THB-M002, Onset
1	Ts	5 cm under soil surface	S-TMB-M002, Onset
1	soil moisture	5 cm under soil surface	S-SMC-M005, Onset
Intensive measurement site (5 sites)^b^			
2	Ta, RH	50 cm above soil surface	HygroClip 2, Rotronic
1	T_litter_, RH	0.5 cm (on soil surface)	HygroClip 2, Rotronic
4	Ts (at 4 spots)	5 cm under soil surface	SKTS 200, Skye
1	soil moisture	5 cm under soil surface	Hydra Probe II, Stevens
1	Ta, RH	2 m (inside the forest)	HygroClip 2, Rotronic
1	Ta, RH, SR, W, prec, BP	2 m (outside the forest)	Weather Transmitter WXT520, Vaisala

^a^Logger: HOBO Micro Station Data Logger H21–002, Onset Computer Corporation

^b^Logger: SDI-Log40 Data Logger, UP GmbH

*Abbreviations*: *BP* barometric pressure, *prec* precipitation, *Ta* air temperature, *Ts* soil temperature, *RH* relative humidity, *SR* solar radiation, *W* wind speed/direction

At each station type, all sensors measured the microclimatic parameters every 10 s (Table 2). The logger calculated and recorded the mean value of these recordings every 10 min. Thus, resulting raw data have a resolution of 10 min.

Data used in this study were measured at the intensive measurement site “Auwald” (AW), a mixed deciduous forest located at 111 m a.s.l. 49.1338 N, 8.3765E) near the river Rhine at Karlsruhe. Its forest inner climate was characterized by an annual mean air temperature of 10.1 °C in 2013 and 11.7 °C in 2014, an annual mean relative humidity of 90% (2013) and 88% (2014) and an annual mean saturation deficit of 1.4 (2013) and 1.9 (2014) measured at 50 cm height inside the forest. Mean values were calculated from the continuous measurements (as hourly mean values) for comparison with data from official weather stations from the German Weather Service (DWD) and annual means. Saturation deficit was calculated according to the Magnus formula over liquid water [36] using temperature (T) and relative humidity (RH) data:

1. E = 6.1*10^((7.5*T)/(T + 237.2))
2. e = E*RH/100
3. SD = E – e

with the saturation vapour pressure E (in hPa), the dew-point temperature e (in °C) and the saturation deficit SD.

One major demand of our study concept was a high comparability of the microclimatic data measured at the different study sites. Therefore, all sensors were calibrated before operation. The combined air temperature and relative humidity sensors were placed in a white protective-shield and fastened at 50 cm height on a wooden post (Fig. 3). For a high comparability of conditions measured at 50 cm height over all study sites, the post was located as far as possible from any trees or shrubs.

**Fig. 5:**
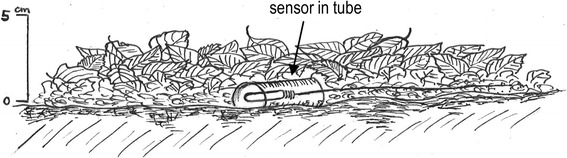
Measurement equipment operated in the litter layer. A combi-sensor for temperature and relative humidity was placed within a small tube and located directly on the ground beneath the litter body

No standard instruments exist to measure conditions in the litter layer. Therefore, a specific measurement device had to be constructed. Standard combi-sensors for air temperature and relative humidity measured in a small protective tube (10 cm length, 2 cm height), which was open to gas transmission at the end and to the ground (Fig. 5). The tube was fixed at a wooden post directly at the soil surface but beneath the litter layer. We defined “litter layer” as the body of newly shed, not (soil science: L-layer) or partly decomposed (Of-layer) organic substances that were hardly mixed with mineral compounds or fine roots. The “humus (layer)” included organic substances on (L−/Of-) and within (Oh-layer) the upper mineral soil layer. The underlying layer, which contained mainly mineral compounds and only small fragments of organic substances, was the first mineral soil layer (Ah) [37].

The sensors for the measurement of soil temperature and soil moisture were placed in the mineral soil (soil.s.: Ah) beneath the humus at about 5 cm depth. The high proportion of enclosed air in the upper, organic rich soil layer (part of the humus body, Oh) would interfere with the technical requirement of close contact between the soil body and the sensors, preventing accurate measurement.

#### Dealing with data gaps

Due to technical problems, data gaps ranging from several days to weeks occurred in some data sets. At the study site AW, for example, no data exists from 20th November to 10th December 2013. To replace these data, a simple linear regression was calculated using daily mean values of temporally overlapping data sets from this station and another forest station. This model was used to calculate missing daily mean values from AW for the duration of the data gap. The best substitution station was evaluated by a cross-correlation analysis.

#### Data processing and analysis

All data were recorded and further processed using Microsoft Excel 2007. Descriptive statistics such as scatter- or box-plots and the calculation of root mean square errors were carried out using R version 3.0.1 [38].

### Official weather stations (in vicinity of study sites)

The measurement of near-ground meteorological data is standardized via the World Meteorological Organization Guidelines, so that data derived from official meteorological (weather) stations are always characterized by open-land measures, as far away as possible from trees, at 1.2–2 m height [27].

**Fig. 6:**
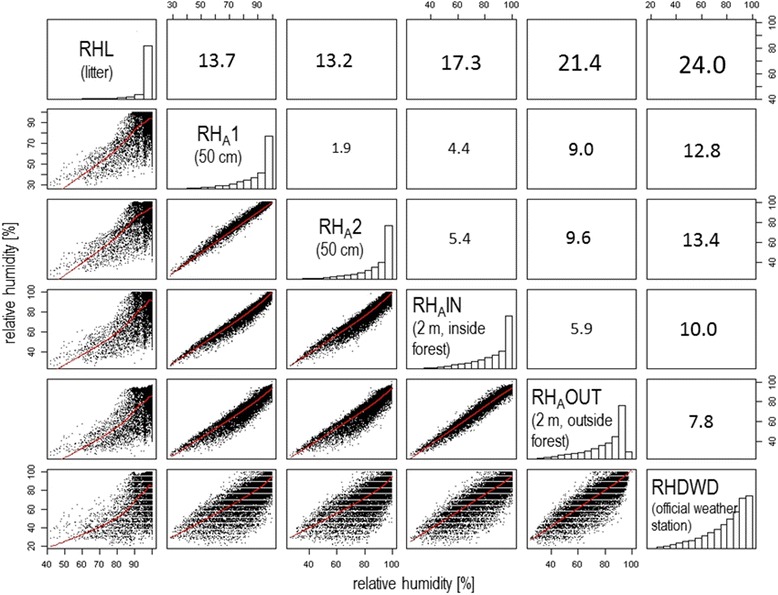
Variability of relative humidity conditions at different measurement points and an official weather station. Scatterplot matrix of hourly relative humidity data measured in the litter (RHL), 50 cm height (RHa1 and 2), 2 m height inside (RHaIN) and outside (RHaOUT) the forest AW and of a nearby official weather station (RHDWD), from 25 October 2012 to 1 January 2015. The lower triangle illustrates scatterplots with LOWESS smoothed lines, the diagonal shows histograms and the upper triangle shows the root mean square error (standard deviation) between data from different measuring points. Relative humidity conditions measured at the official weather station were on average 24% RH lower (thus drier) than those measured in the litter layer

**Fig. 7:**
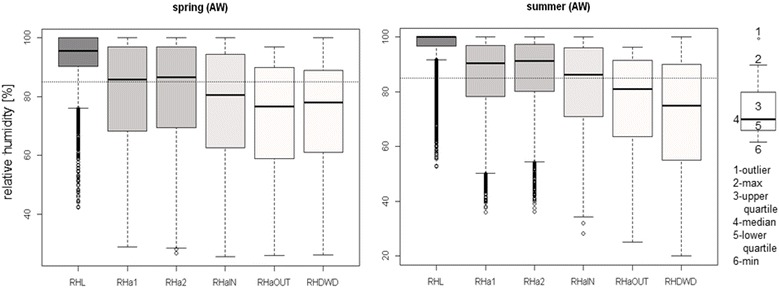
Seasonal character of relative humidity conditions and implications for *I. ricinus* water balance. Boxplots illustrate hourly data measured in spring (March to May) and summer (June to August) 2013 and 2014 at the same measuring points as in Fig. 6. There is a strong deviation between conditions in the litter layer and conditions at 50 cm height above and to data from the official weather station. The horizontal dotted line depicts the critical equilibrium humidity for *Ixodes ricinus* of 85% RH [14, 19]

The official weather station used for comparisons in this report (Figs. 1c, 2b, 6, 7) is operated by the German Weather Service, and is named “Rheinstetten”  (ID: 04177). It is located at 48°58′N, 8°20′E at an altitude of 116 m a.s.l. It is located in the open-land at 2 m height above the ground [39]. Its relative humidity data were used for comparisons with our microclimatic station AW in a resolution of daily means (Figs. 1, 2) or hourly means (Figs. 6, 7).

## Results

### In situ meteorological measurements *versus* official weather data (spatial measurement error)

Fig. 6 illustrates the variance of measured, hourly data for relative humidity at different points in the study site AW and the official weather station “Rheinstetten” from 25th October 2012 to 1st January 2015.

The data indicate that humidity conditions in the litter layer (RHL), the place where ticks rest, develop and regain water losses, differ strongly from all other measuring points (scatterplots: first column from left, RMSE: first line from top). Conditions in the litter were moist, generally with values of RH between 90 and 100%. The relative humidity in the lower atmosphere of the forest (50 cm and 2 m) ranged between about 30–100%. Looking at the root mean square errors (RMSE), conditions in the litter layer were on average about 13–24% RH moister than at all other measuring points.

In contrast, data from the official weather station (RHDWD) were on average about 8–24% RH drier. Conditions dried down to 20% RH and showed a characteristic deviation from linearity, forming a belly to minor relative humidity values, compared to all other measuring points.

If water evaporates from the humus and soil body, the added gaseous water should increase the absolute and relative humidity of the surrounding air masses. In addition, reduced turbulences caused by the forest canopy should lead to a clear decrease in humidity with increasing distance from the ground (where water evaporates) and from inside to outside the forest. The results showed that at all intensive study sites, relative humidity conditions were moistest in the litter, followed by the air in 50 cm, by 2 m in the forest and, characterized by driest conditions, 2 m outside the forest (Table 3).

**Table 3:** Annual averages of RH (%) at different microhabitats of the five intensive study sites

Site name	Altitude (m)^a^	RHL: Litter layer	RHa1: 50 cm	Rha2: 50 cm	RHaIN: 2 m inside	RHaOUT: 2 m outside	RHDWD: Official weather station
Auwald (AW)	111	96	88	88	85	79	78
Hardtwald (HW)	117	96	85	84	83	77	78
Michaelsberg (MB)	253	97	84	84	83	74	78
Schwarzwald (SW)	610	96	80	80	77	68	81
Drackenstein (DS)	755	97	87	85	83	77	83
Mean		96	85	84	82	75	80

^**a**^Metres above sea level

Figure 7 illustrates conditions measured in, and close to, the forest AW and those measured at a nearby official weather station. Illustrated data comprise spring, the season of highest tick activity, and summer, the season during which lack of moisture is expected to be most critical for tick survival. Relative humidity conditions in the litter layer (RHL) were constantly high. For more than three quarters of the time, RH stayed over 85% so that ticks were able to regain water losses (dotted, horizontal line), especially in summer*.* Even in the zone of active tick questing at 50 cm height (labeled as RHa1 and RHa2), RH stayed over 85% half of the time in spring and even more often in summer. With increasing distance from the forest ground (RHaIN, 2 m), or, even more, from the forest itself (RHaOUT, RHDWD), the humidity conditions became drier. Outside of the forest, relative humidity rarely reached levels exceeding 90%.

Our results clearly underline the theoretical considerations about the error sources. The error induced by environmental differences between the in situ measurements and the official weather stations would lead to a substantial misinterpretation of the results.

### Continuous *versus* occasional measurements (temporal measurement error)

**Fig. 8:**
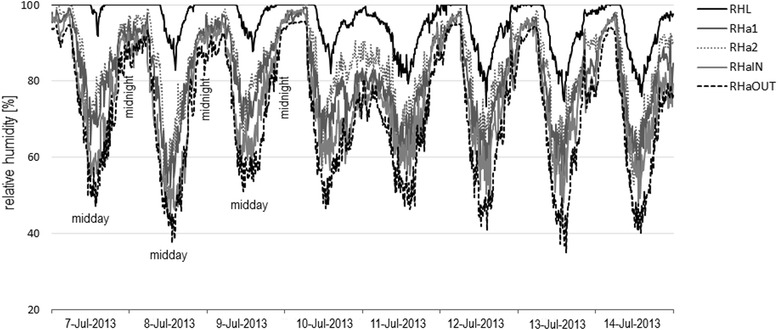
Course of relative humidity conditions depending on time of day. The time series illustrates the range of RH conditions recorded one week in July 2013 at AW. As with increasing temperatures during the day (not shown), relative humidity values drop and rise along with cooling at night. Occasional measurements, once per day, would lead to a bias when compared to the range of actual conditions

The analysis in this subsection focus on the evaluation of potential temporal measurement errors due to occasional measurements (Fig. 2c). Fig. 8 illustrates the relative humidity conditions recorded as 10 min averaged values at different points of the study site AW. A period of one week in July 2013 is enough to demonstrate the strong diurnal periodicity in relative humidity conditions, with maximum values at night and minimum values during the day. Measurements only taken occasionally over time fail to record this behavior.

In addition, Table 4 depicts the effect of occasional measurements using the example of mean values of this 7-day recording period, when data are measured only once per day at a specific time. On an averaged basis, measurements at midday recorded 13–14% drier conditions than measurements in the morning or afternoon, and 25% RH drier conditions than at midnight. Hence, for example, using data recorded once per day at midday would lead to a serious underestimation of the actual relative humidity. Thus, the time of day for the occasional recording is one source of temporal measuring error.

**Table 4:** RH conditions at different day times and measuring points in July 2013 in Auwald

	Midnight	9:00 am	Midday	6:00 pm	7 day Min	7 day Max
RHL	100	97	88	95	73	100
RHa1	91	76	67	81	46	99
RHa2	94	85	67	83	48	100
RHaIN	88	70	60	73	42	99
RHaOUT	86	65	49	68	35	96
Mean	91	79	66	80	–	–

## Discussion

The results generally reaffirm the need for additional measurements in forests as soon as climatic statements and analysis are not restricted to open-land areas [3, 11]. In addition, they confirm a strong spatial measuring error between climatic conditions measured in forests and at official meteorological stations [3]. The strong deviations are very likely due to a combination of habitat type and structure (forest *versus* open land), horizontal distance (between study site and location of meteorological station) and vertical distance (measurement in 2 m height vs near-ground). Both the habitat type and structure, as well as the vertical distance from the absorbing surface, directly influences the impact of solar radiation, and the resulting radiation induces evaporation and heating processes [40]; therefore, these are expected to be the main drivers explaining observed differences. One aspect is the reduction of incoming solar radiation by the forest canopy and the associated reduction in warming and dehydration of the ground compared to open land.

As expected, our data indicate that the moistest conditions occur in the litter layer, followed by the near-ground at 50 cm height. A litter body has a specific capacity to store water from precipitation (interception storage capacity) and to retain water from evaporation, which increases with both litter thickness and mass [41, 42]. Thus, even if humidity conditions in the atmosphere are relatively low, the litter layer has its own capacity to stay moist over a longer time and to increase humidity conditions in the near-ground air masses, as shown by our data.

As mentioned previously, laboratory experiments showed that *Ixodes ricinus* has an equilibrium humidity of 85% RH, which means ticks desiccate if conditions fall below this threshold, although their body water level remains stable and water losses can be replenished actively (with energy losses) if conditions are moister than 80–85% RH [14, 19]. Each forest microhabitat offers its own specific humidity conditions and data measured outside of the forest do not represent these conditions (Figs. 6, 7). By using official weather data (RHDWD), for example, one would conclude that ticks were mainly exposed to desiccating conditions and predict a high mortality. In contrast, microclimatic data measured in the litter layer indicate a local, continuous and sufficient moisture supply for ticks. Even at 50 cm height, actively questing ticks can maintain their body water level half of the time, or even more in summer. It should be noted that, in this discussion, the focus is on the representativeness of the data to capture the meteorological conditions for a specific species. Data from official weather stations can be suitable if used for an open-land species, but not for forest dwelling (tick) species.

However, even if climatic conditions measured directly within the ticks microhabitat provide a better approximation than those from open-land stations, there is still a need for more information about the conditions to be found, for example, in the first millimeter of an evaporating plant leaf or moist plant litter, where *I. ricinus* is actually located. It is possible that small, unengorged ticks pressing on a moist or evaporating surface experiences more suitable humidity conditions than our measuring approach indicates.

Our results also demonstrate that forest measurements do not represent the microclimatic conditions ticks are naturally permanently exposed to if data are only occasionally measured. Measurements once a day result in serious temporal measuring errors, lacking information of maximum or minimum values and summation effects. If a specific time of day is preferred for measuring, results can be strongly biased towards one end of the range of conditions, with resulting misinterpretations regarding to the tick’s ecology.

The measuring setup to record such detailed microclimatic data in forests is, however, costly. The initial acquisition of equipment requires knowledge of the technique used and is time-consuming in operation and processing of the data. For example, it is necessary to protect the devices in the field from damage. This applies, on the one hand, to weather events such as showers or snow, for which extra housings for loggers were necessary. To avoid damage by game animals and rodents, cables need to be protected in an additional plastic cable protector and ground cables must be buried from the mast to the sensor about 5 cm deep. Operation in the field includes regular visits to exchange batteries and for data read out. An advantage of official measuring stations, whose data output has already been checked by experts for quality and completeness, is not possible with the use of self-obtained data.

This raises the question, as to which kind of research questions require such extensive measurements. This is certainly dependent on the scale at which we want to look at something [2]. To model on a larger spatial scale, for example the landscape scale, we need extrapolated and gridded data that cover the total model area. Interestingly, such data outperformed the prediction power of our locally measured data when modelling tick density distribution on an annual averaged basis [24]. Possible reasons are that gridded data represent processes leading to tick density pattern better on a large temporal scale [1], or it is possibly an artefact of the restricted number of data points.

To explain the pattern on a habitat scale, which means to picture microclimatic mechanisms shaping (questing) tick abundances in single habitats, the large discrepancies between official weather data and microclimatic data strongly recommend the use of microclimatic in situ measurements. If this is not the case, only weak, non-significant correlations are expected [11, 43].

Referring to Table 1, use and description of weather data in tick research is done in various ways hindering further comparisons of research results. Therefore, the following proposes a list of useful information to be given in the meteorological methods section in future:

(i) Information about the measuring equipment: (a) Origin (official weather station or in situ measurements); (b) Equipment manufacturer and type of logger and sensors (if measured in situ); (c) Location (latitude and longitude, habitat type, location within or distance from study site); (d) Altitude; (e) Mean annual values of temperature and humidity parameters (to characterize the study site);
(ii) Information about the continuously recorded meteorological data: (a) Recorded parameters and height of (each) sensor; (b) Resolution of raw data (here: 10 min); (c) Resolution of used data in analysis (here: hourly and daily mean values); (d) A detailed explanation of the intension behind the database used: What is captured with this climatic data? Why this resolution?

Doing this, we expect better comparisons and thus important findings about the mechanisms underlying the activity and density pattern of ticks, which is the key to prediction in the future [2].

## Conclusions

To date, research on the influence of climatic factors on *I. ricinus* ticks still uses a variety of data acquisition and processing methods (Table 1). Data from our measuring setup clearly show the large, statistical deviation from conditions measured in a forest habitat and data derived from nearby official weather stations. This high spatial measuring error effects every statistical correlation between tick data sampled in forests and weather data from open-land stations, and the conclusions drawn with respect to underlying mechanisms. In addition, there is a high temporal measuring error associated with occasional measurements, due to the lack of important information on temporal variability. We therefore highly recommend continuous measurements of meteorological data directly in the species’ microhabitat. In addition, methodological information should be reported uniformly. We therefore proposed a list of information to be placed in the methods section, which seems helpful to improve the comparability of tick research concerning weather data in the future. However, important questions remain unanswered: what kind of research questions and parameters necessarily need such an accurate climatic dataset? Are there research questions that official weather data are sufficient for? What kind of tick data are needed for such an accurate climatic dataset - is monthly tick sampling sufficient? What mechanisms can we investigate by the use of different data level combinations?

## Abbreviations

RH: Relative humidity (%)
SD: Saturation deficit (hPa)
T_A_: Air temperature (°C)
T_S_: Soil temperature (°C)
